# Transcriptomic clues to understand the growth of *Lactobacillus rhamnosus* in cheese

**DOI:** 10.1186/1471-2180-14-28

**Published:** 2014-02-07

**Authors:** Camilla Lazzi, Silvia Turroni, Andrea Mancini, Elisa Sgarbi, Erasmo Neviani, Patrizia Brigidi, Monica Gatti

**Affiliations:** 1 Department of Food Science, Parma University, Parco Area delle Scienze 48/A, 43124 Parma, Italy; 2 Department of Pharmacy and Biotechnology, University of Bologna, Via Belmeloro 6, 40126 Bologna, Italy; 3 Current address: Nutrition and Nutrigenomics Group, Department of Food Quality and Nutrition, Research and Innovation Centre, Fondazione Edmund Mach di San Michele all’Adige, Trento, Italy

**Keywords:** *Lactobacillus rhamnosus*, Cheese, cDNA-amplified fragment length polymorphism, Quantitative real-time reverse transcription-PCR

## Abstract

**Background:**

*Lactobacillus rhamnosus* is a non-starter lactic acid bacterium that plays a significant role during cheese ripening, leading to the formation of flavor. In long-ripened cheeses it persists throughout the whole time of ripening due to its capacity to adapt to changing environmental conditions. The versatile adaptability of *L. rhamnosus* to different ecosystems has been associated with the capacity to use non-conventional energy sources, regulating different metabolic pathways. However, the molecular mechanisms allowing the growth of *L. rhamnosus* in the cheese dairy environment are still poorly understood. The aim of the present study was to identify genes potentially contributing to the growth ability of *L. rhamnosus* PR1019 in cheese-like medium (CB) using a transcriptomic approach, based on cDNA-amplified fragment length polymorphism (cDNA-AFLP) and quantitative real-time reverse transcription-PCR (qPCR).

**Results:**

Using three primer combinations, a total of 89 and 98 transcript-derived fragments were obtained for *L. rhamnosus* PR1019 grown in commercial MRS medium and CB, respectively. The cDNA-AFLP results were validated on selected regulated genes by qPCR. In order to investigate the main adaptations to growth in a cheese-mimicking system, we focused on 20 transcripts over-expressed in CB with respect to MRS. It is worth noting the presence of transcripts involved in the degradation of pyruvate and ribose. Pyruvate is a intracellular metabolite that can be produced through different metabolic routes starting from the carbon sources present in cheese, and can be released in the cheese matrix with the starter lysis. Similarly the ribonucleosides released with starter lysis could deliver ribose that represents a fermentable carbohydrate in environments, such as cheese, where free carbohydrates are lacking.

Both pyruvate degradation and ribose catabolism induce a metabolite flux toward acetate, coupled with ATP production via acetate kinase. Taking into account these considerations, we suggest that the energy produced through these pathways may concur to explain the great ability of *L. rhamnosus* PR1019 to grow on CB.

**Conclusions:**

By a transcriptomic approach we identified a set of genes involved in alternative metabolic pathways in *L. rhamnosus* that could be responsible for *L. rhamnosus* growth in cheese during ripening.

## Background

*Lactobacillus rhamnosus* is a facultatively heterofermentative Lactic Acid Bacterium (LAB) frequently encountered in many dairy products, where it can be added as a probiotic microorganism or can be naturally present arising from raw milk. LAB may play different roles in cheese manufacture: some species participate in the fermentation process and contribute to acid production acting as starter LAB (SLAB), whereas others, called non-starter LAB (NSLAB), are mainly implicated in the maturation process. In particular, *L. rhamnosus* plays a significant role during ripening, leading to the formation of flavor [1,2] and, for this reason, members of this species are generally recognized as NSLAB. It is noteworthy that NSLAB generally have a high tolerance to hostile environments, such as those with high salt concentration, low moisture, 4.9-5.3 pH values, low temperatures and deficiency of nutrients [3-5]. Moreover, several studies have reported that in long-ripened cheese varieties, NSLAB populations dominate during aging after SLAB decline due to autolysis [6,7]. Increasing by about four to five orders of magnitude within a few months, NSLAB can have a major impact in determining curd maturation and final characteristics of cheese [5]. In particular, *L. rhamnosus* has been shown to become dominant within NSLAB population in several cheeses, including Parmigiano Reggiano (PR) [8,9]. It persists throughout the whole time of PR cheese ripening (1 to 20 months) and this implies its capacity to adapt to changing environmental conditions [10]. Notably, different *L. rhamnosus* strains have been detected in relation to specific steps of cheese ripening, suggesting that these strains may have specific metabolic activities, which could account for the adaptation to the changing microenvironment of cheese ripening [11].

Up to date, the molecular mechanisms allowing the growth of *L. rhamnosus* in cheese are still poorly understood. NSLAB development during ripening can be attributed to their ability to use the major nutrient sources available in ripened cheese, that is lactose-free. The nutrient sources available include milk components modified by technological treatment (rennet addition and curd cooking) and starter LAB development, starter LAB metabolites and cell lysis products. Potential substrate for microbial growth are represented by small peptides or amino acids [4], citrate, lactate, and free fatty acids [12]. Additionally, sugars and phospholipids, nucleic acids and peptides can be released in the cheese matrix when SLAB autolysis starts to occur [13-15]. These compounds represent carbon sources that could yield intracellular pyruvate (i.e. through metabolism of citrate, lactate, amino acids, and nucleotides) or be converted into different metabolites.

To investigate the metabolic pathways occurring in *L. rhamnosus* during cheese ripening, Bove and colleagues [16] recently compared the proteomic profiles of 10 *L. rhamnosus* strains grown in MRS and a cheese-like medium (Cheese Broth, CB). Differently from MRS, which is the standard laboratory medium for lactobacilli [17] and is considered to be a rich substrate with glucose as the primary carbon source for microbial growth, CB is an experimental medium formulated with 20-month-ripened PR cheese [10,16,18], that tries to mimic the nutritional composition of PR cheese during ripening. PR cheese, and thus CB, is considered to be a substrate poor in carbohydrates and characterized by the absence of milk sugar, lactose. During the curd acidification step of PR cheese production, the conversion of lactose into lactic acid is the main biochemical process that occurs. Lactose is completely depleted within 24 to 48 h [12]. The composition of CB, prepared as the protocol of Neviani et al. [10], is the following (g/l): proteins, 80.92; lactose, 0.00; glucose, 0.00; galactose, 0.00; lactic acid, 3.82; NaCl, 3.4; sodium citrate, 20.64.

According to the findings of Bove et al. [16] compared to the cultivation in MRS, the differentially expressed proteins under cheese-like conditions were mainly linked to protein biosynthesis and catabolism, nucleotide and carbohydrate metabolisms, citrate metabolism, cell wall and exopolysaccharide biosynthesis, cell regulation, oxidation/reduction processes, and stress response [16]. Notably, *L. rhamnosus* produced lactic acid as the primary end product when growing in MRS, whereas in CB low levels of lactic acid together with high levels of acetic acid were detected for all strains. Despite this common trend, the authors also observed strain-specific physiological responses, suggesting a strain variability in the adaptation to changing environmental conditions in accordance with genetic polymorphism studies [11]. Among all strains, *L. rhamnosus* PR1019, isolated from PR 4-month-ripened cheese, has shown the greatest ability to growth in CB reaching after 48 h the highest cell numbers with one of the lowest levels of lactic acid and one of the highest levels of acetic acid.

For this reason, in the present work, we focused our attention only on this strain, with the aim to identify the genes that could concur to explain its growth ability in CB and its acid acetic production. The physiological adaptation of *L. rhamnosus* PR1019 in CB was evaluated using a transcriptomic approach, based on cDNA-amplified fragment length polymorphism (cDNA-AFLP) and quantitative real-time reverse transcription-PCR (qPCR). cDNA-AFLP is one of the most robust and sensitive transcriptomic technologies for genome-wide expression studies, with the main advantage of not requiring any prior knowledge of gene sequences while allowing the detection of lowly expressed genes through transcript amplification [19].

Using this approach, we identified a set of genes resulted over-expressed in CB compared to MRS, potentially involved in alternative metabolic pathways. Interesting genes were searched in other NSLAB and SLAB genomes with the aim to explore their diversity. Overall, the results described in this work highlight mechanisms of adaptation leading to the production of acetic acid coupled with ATP generation, that could support the *L. rhamnosus* growth in cheese during ripening.

## Methods

### Bacterial growth conditions

*L. rhamnosus* PR1019 was isolated from Parmigiano Reggiano (PR) at 4 months of ripening on cheese based medium [10] plate counts and identified by 16S rDNA gene sequencing [11] and species-specific PCR [20]. The strain was cultivated in MRS broth (Oxoid) or Cheese Broth (CB) at 30°C, under anaerobiosis, for 24 or 48 h, respectively. CB, a culture medium that mimics raw-milk long-ripened cheese, was prepared according to the modified protocol described by Bove et al. [16,18].

### RNA extraction and cDNA synthesis

The growth of *L. rhamnosus* PR1019 in MRS and CB broth was monitored by measuring optical density (OD) at 600 nm. About 10^9^ cells at the top of logarithmic phase were harvested, and total RNA, stabilized with RNAprotect Bacteria Reagent (QIAGEN), was isolated using RNeasy Protect Bacteria Mini Kit (QIAGEN). Three independent biological experiment were made. RNA was quantified using a NanoDrop ND-1000 spectrophotometer (NanoDrop Technologies) and visualized by formaldehyde agarose gel electrophoresis according to standard procedures. All RNAs were of sufficient quantity (>350 ng/μl) and high quality (A260/A280 ratio 2.0 to 2.1). After a step of mRNA enrichment and polyadenylation of RNA transcripts, cDNA was synthesized by reverse transcription (RT) using a biotinylated oligo (dT), following the protocol reported by Bove et al. [18]. For qPCR, single-stranded cDNA was prepared in a final volume of 20 μl from total RNA using an oligo (dT) primer and SuperScript™ II reverse transcriptase (Invitrogen), according to the manufacturer’s instructions. Briefly, 12-μl reaction mixtures containing 500 ng of oligo (dT) primer, 2 μg total RNA and 10 nmol dNTP mix in DEPC-treated H_2_O were heated to 65°C for 5 min, added with 4 μl of 5X First-Strand Buffer (Invitrogen) and 200 nmol DTT, and then incubated at 42°C for 2 min. RT reactions were started by the addition of 200 U of enzyme, incubated at 42°C for 50 min and inactivated by heating at 70°C for 15 min. RT step was carried out in duplicate.

### cDNA-AFLP

cDNA-AFLP analysis was carried out as described by Bove et al. [18]. The protocol is based on the production of cDNA-AFLP fragments that are detected using infrared dye (IRD) detection technology and the Odyssey Infrared Imaging System. Briefly, after cDNA synthesis, a double digestion was carried out with EcoRI and MseI restriction enzymes and fragments were captured with the aid of streptavidin-coated magnetic beads. Digested cDNA fragments were subsequently ligated with adaptors to allow selective amplification with EcoRI primers labeled with an infrared dye (IRDye™ 700 phosphoramidite), and unlabeled MseI-N (Eurofins MWG Operon). Three primer combinations were used to selectively amplify the expressed genes: DY-EcoRI-AC/MseI-AT, DY-EcoRI-AT/MseI-AC and DY-EcoRI-AT/MseI-AT [18]. Ligators and primers used are reported in Table 1. Separation of cDNA-AFLP fragments was carried out in a polyacrylamide gel and visualized by Odissey (LI-COR Biosciences) at 700 nm.

**Table 1:** Primer and adaptor sequences

**Primer/adaptor**	**Sequence (5′-3′)**	**Application**
Adaptor EcoRI-f	CTCGTAGACTGCGTACC	Ligation
Adaptor EcoRI-r	AATTGGTACGCAGTCTAC	Ligation
Adaptor MseI-f	GACGATGAGTCCTGAG	Ligation
Adaptor MseI-r	TACTCAGGACTCAT	Ligation
EcoRI-0	GACTGCGTACCAATTC	Non-selective PCR
MseI-0	GATGAGTCCTGAGTAA	Non-selective PCR
5′DY-EcoRI-AT	GACTGCGTACCAATTCAT	Selective PCR
5′DY-EcoRI-AC	GACTGCGTACCAATTCAC	Selective PCR
MseI-AT	GATGAGTCCTGAGTAAAT	Selective PCR
MseI-AC	GATGAGTCCTGAGTAAAC	Selective PCR
EcoRI-AC	GACTGCGTACCAATTCAC	Re-amplification PCR
EcoRI-AT	GACTGCGTACCAATTCAT	Re-amplification PCR

Primer sets were designed as reported by Bove et al. [18].

### cDNA-AFLP fragment isolation, re-amplification and sequencing

Transcript-derived fragments (TDFs) of interest were cut from polyacrylamide gels as reported by Vuylsteke et al. [19], resuspended in 100 μl of distilled water and subsequently re-amplified using the re-amplification and selective PCR primers EcoRI-AC/MseI-AT, EcoRI-AT/MseI-AC and EcoRI-AT/MseI-AT (Table 1) according to the origin of cDNA-AFLP fragments. Amplification reactions were performed in a final volume of 50 μl containing 13 μl of resuspended DNA fragment, 25 mM MgCl_2_, 10X PCR buffer, 2 μM EcoRI-N primer, 2 μM MseI-N primer, 5 mM dNTPs, 0.5 μl of AmpliTaq 360 DNA polymerase (5U/μl) and 2 μl of 360 GC enhancer (Applied Biosystems-Life Technologies). PCR consisted of: i) 30 s of denaturation step at 94°C, 30 s of annealing step at 65°C (reduced of 0.7°C at each cycle) and 1 min of extension step at 72°C for 13 total cycles; ii) 10 s of denaturation step at 94°C, 30 s of annealing step at 56°C and 1 min of extension step at 72°C (extended for 1 min at each cycle) for 35 total cycles. These cycles were preceded by a common denaturation step of 2 min at 94°C and followed by a final 10-min extension at 72°C, and were carried out in a Mastercycler ep gradient S thermal cycler (Eppendorf). Amplified products were checked on a 1% agarose gel with a 100-bp marker (Invitrogen) and subsequently purified using the Wizard® SV Gel and PCR Clean-Up System according to the manufacturer’s instructions (Promega Corporation). Amplified fragments were then cloned in *E.coli* using the pGEM-T Easy Vector System kit (Promega Corporation), and plasmids from selected clones were purified using PureYield MiniPrep System kit (Promega Corporation) referring to the producer’s manual. Cloned fragments were finally sequenced by Eurofins MWG Operon using primers M13 and sequences were analysed by BLAST alignment [21]. TDF sequences were deposited in the DDBJ database under the accession numbers AB896768 to AB896786.

### qPCR and data processing

qPCR was carried out using the LightCycler SYBR Green system (Roche) as previously described [22]. Briefly, 1 μl of cDNA template was used in each reaction along with 4 μl of SYBR Green PCR master mix (Roche) and 10 pmol of the appropriate gene-specific primers in a final volume of 20 μl. The following cycle profile was used: 10 min at 95°C, 40 repeats of 15 s at 95°C, 25 s at 58°C for *spxB*, *ulaE* and *16S rDNA* genes or 55°C for *xfp*, 72°C for 20 s (30 s for *16S rDNA*) and an additional 5-s incubation step at 81°C for fluorescence acquisition. Oligonucleotide sequence information and detailed primer-specific conditions are given in Table 2. Two technical replicates were done for each combination of cDNA and primer pair. To assess background and residual DNA contamination, a no-template control (NTC) and a no-reverse transcription control (NoRT) were performed for each target. DNA contamination was considered to be negligible when the difference in C_q_ (quantification cycle) between the sample and the respective NoRT was above 5 cycles. Product detection and PCR specificity were checked post-amplification by examining the dissociation curves. PCR amplicons were resolved by 2% agarose gel electrophoresis to verify the expected size. To evaluate repeatability and reproducibility of the qPCR assay, intra- and inter-assay coefficients of variation (CV) were assessed. The intra-assay CV was from 0.7 to 7.6% whereas the inter-assay CV ranged from 8.3 to 18.8%. Amplification efficiency was calculated from the slope of standard curves generated with two-fold serial dilutions of the same cDNA sample, as E = 10^(-1/slope)^. Relative expression of target genes was determined using the ΔΔ*C*_T_ method after Pfaffl correction [23]. *16S rDNA* was used as a reference gene.

**Table 2:** qPCR settings and relative transcript abundance

**Target gene**	**Primer sequence (5′-3′)**^**a**^	**Product size (bp)**	**PCR efficiency**	**Expression ratio**^**b**^
*spxB*	fwd: TACCGGAAACTGCTTGGTATC	155	1.93	8.97
	rev: CTGGAAAACCGCATCTTTGT			
*ulaE*	fwd: CACTAGCCAAATCAATCGCC	90	2.05	5.78
	rev: GCCATCGTCGGTTTCCATTA			
*xfp*	fwd: CGTGAAGAAGGCGATATC	215	2.01	5.98
	rev: TTCCAAGTCCACTCCTGA			
*16S rDNA*	fwd: GCYTAACACATGCAAGTCGA	500	1.85	/
	rev: GTATTACCGCGGCTGCTGG			

^a^Primer sets were designed based on the sequences of cDNA-AFLP fragments. Primers for *16S rDNA* gene were designed as reported by Giraffa et al. [24].

^b^Target gene expression was calculated relative to *16S rDNA* as a reference gene using the efficiency-corrected ΔΔ*C*_T_ method [23]. The relative expression ratios in CB compared to MRS are shown.

### *In silico* analysis

TDF sequences were annotated using BLAST search. Pathway assignment was performed according to COG (Cluster of Orthologous Groups) [25] functional categories and KEGG (Kyoto Encyclopedia of Genes and Genome) [26] pathway database. Gene synteny across NSLAB and SLAB genomes was explored through the web server SyntTax [27]. Genome mining for promoter and terminator elements was performed using PePPER toolbox [28]. Translated protein sequences were subjected to Pfam motif analysis [29]. Protein alignments were performed using ClustalW2 [30] and used for phylogenetic tree construction at the Interactive Tree of Life [31]. Multisequence amino acid alignments were represented using CLC-Bio sequence viewer [32].

## Results and discussion

### cDNA-AFLP analysis

In this study, the cDNA-AFLP technique [18] was applied to profile the transcriptome of a *L. rhamnosus* strain grown in conditions mimicking cheese ripening. Despite it is not widely used in bacteria, cDNA-AFLP can be considered an ideal system for genome-wide expression analysis, mainly for the detection of lowly expressed genes.

**Figure 1:**
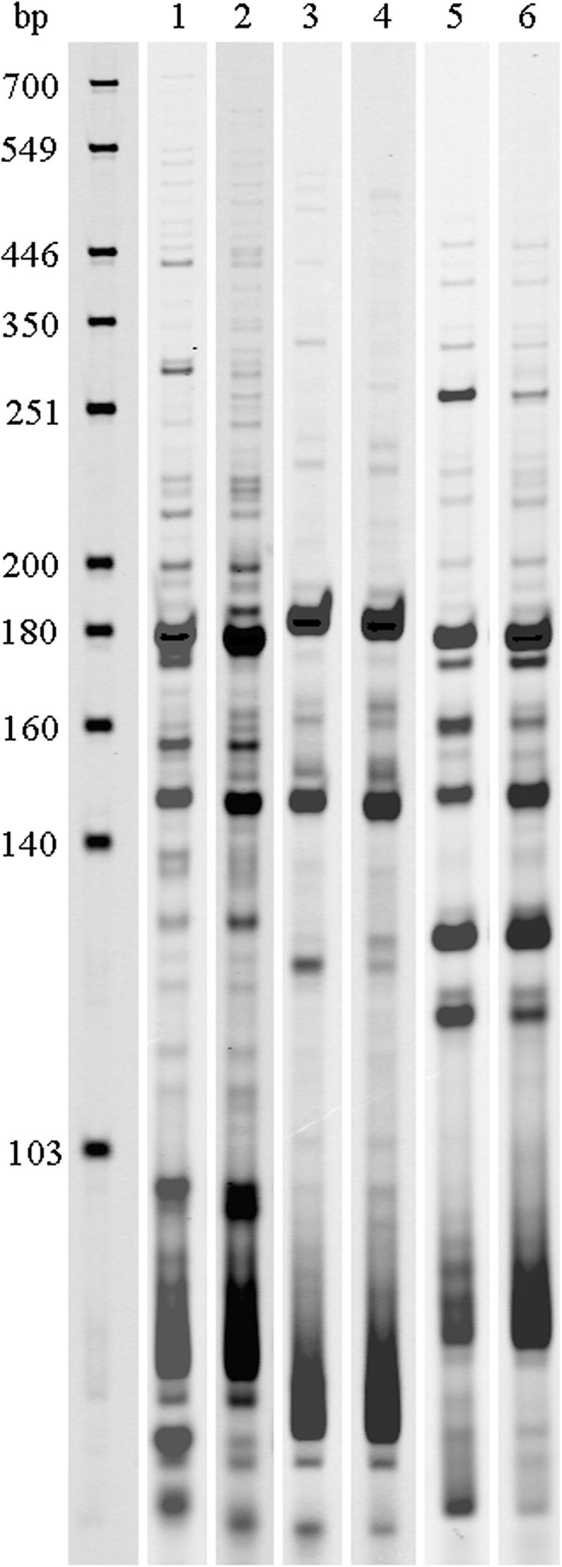
**cDNA-AFLP fingerprint of *L. rhamnosus *PR1019 grown in MRS and CB, obtained with three different primer combinations.** M, 50–700 bp IRDye700 Sizing Standard; lanes 1, 3 and 5, cDNA-AFLP fingerprinting of *L. rhamnosus* cultured in MRS using EcoRI-AC/MseI-AT, EcoRI-AT/MseI-AC and EcoRI-AT/MseI-AT primer combination, respectively; lanes 2, 4 and 6, cDNA-AFLP fingerprinting of *L. rhamnosus* cultured in CB using EcoRI-AC/MseI-AT, EcoRI-AT/MseI-AC and EcoRI-AT/MseI-AT primer combination, respectively.

Three primer combinations were used to selectively amplify the genes expressed by *L. rhamnosus* PR1019 in CB and MRS, allowing to generate different cDNA-AFLP profiles with a fragment size ranging from 50 to 500 bp (Figure 1). A total of 89 and 98 TDFs were detected in MRS and CB, respectively. In order to investigate the main adaptations of *L. rhamnosus* to the PR cheese environment, we focused on TDFs over-expressed in CB.

### Identification of TDFs over-expressed in CB

Twenty TDFs strongly over-expressed by *L. rhamnosus* in CB compared to MRS were extracted from gel and used as templates for re-amplification by PCR.

Direct sequencing of the PCR products failed probably because the sequences were contaminated with co-migrating bands or were too small (data not shown). Products from bands were then cloned and PCR amplicons from 10 individual colonies were sequenced. Reliable (> 100 bp) sequences were obtained for 19 of the 20 TDFs. Each sequence was identified by similarity search using the BLAST program against the GenBank non-redundant (nr) public sequence database.

As shown in Table 3, 8 transcripts (approximately 42% of selected sequences) showed a significant similarity to sequences with known function, 5 transcripts (26.5% of selected sequences) were closely related to *L. casei* plasmid sequences, 4 transcripts (21% of selected sequences) were annotated as hypothetical protein-coding sequences, and 2 transcripts (10.5% of selected sequences) were identified as 5S rRNA.

**Table 3:** **Transcript-derived fragments (TDFs) from *L. rhamnosus *** **PR1019 over-expressed in CB compared to MRS**

**TDF no**	**Primer combination**	**Length (bp)**	**Biological function**^**a**^	**Organism annotation**^**b**^	**Max identity - E-value**^**c**^	**Accession no.**	**Pathway assignment**^**d**^	
							**COG**^**e**^	**KEGG**
37	AC/AT	396	Guanylate kinase (EC 2.7.4.8)	*L. rhamnosus* GG	98% - 1e-84	YP_003171760.1	COG0194 [F]	ko00230: Purine metabolism
40	AC/AT	302	Putative phosphoketolase (EC 4.1.2.9)	*L. rhamnosus* GG	99% - 3e-57	YP_005864692.1	COG3957 [G]	ko00030: Pentose phosphate pathway
48	AC/AT	199	Monooxygenase	*L. rhamnosus* Lc 705	95% - 6e-17	YP_003174467.1	COG2329: Conserved protein involved in polyketide biosynthesis related to monooxygenase [R]	_
54	AC/AT	137	Hypothetical protein	*L. rhamnosus*	77% - 2e-07	WP_005689523.1	_	_
72	AC/AT	340	Lipoteichoic acid synthase LtaS Type IIa (EC 3.1.6)	*L. rhamnosus* Lc 705	100% - 5e-43	YP_003173514.1	COG1368: Phosphoglycerol transferase and related proteins, alkaline phosphatase superfamily [M]	_
76	AC/AT	433	Conserved hypothetical protein	*L. rhamnosus* Lc 705	85% - 9e-27	YP_003174890.1	_	_
86	AC/AT	109	L-xylulose 5-phosphate 3-epimerase (EC 5.1.3.22)	*L. rhamnosus* GG	94% - 9e-13	YP_003172471.1	COG3623 [G]	ko00040: Pentose and glucuronate interconversions
93	AC/AT	305	Pyruvate oxidase (EC 1.2.3.3)	*L. rhamnosus* GG	93% - 5e-40	YP_003171582.1	COG3961: Pyruvate decarboxylase and related thiamine pyrophosphate-requiring enzymes [G]	ko00620: Pyruvate metabolism
95	AC/AT	229	Plasmid pNCD0151	*L. casei*	98% - 4e-68	Z50861.1	_	_
97	AC/AT	227	Plasmid pNCD0151	*L. casei*	96% - 3e-64	Z50861.1	_	_
106	AC/AT	170	Plasmid pNCD0151	*L. casei*	97% - 9e-48	Z50861.1	_	_
120	AC/AT	107	Hypothetical protein	*L. casei*	96% - 4e-10	WP_003574536.1	_	_
121	AC/AT	105	Imidazoleglycerol-phosphate dehydratase (EC 4.2.1.19)	*L. rhamnosus* Lc 705	92% - 5e-08	YP_003174148.1	COG0131 [E]	ko00340: Hystidine metabolism
122	AC/AT	102	Plasmid pNCD0151	*L. casei*	96% - 5e-23	Z50861.1	_	_
162	AT/AC	350	Calcineurin-like phosphoesterase family protein	*L. rhamnosus* ATCC 8530	97% - 3e-70	YP_005872999.1	COG0737: 5′-nucleotidase/2′,3′-cyclic phosphodiesterase and related esterases [F]	_
168	AT/AC	238	Plasmid pNCD0151	*L. casei*	98% - 1e-68	Z50861.1	_	_
170	AT/AC	222	Hypothetical protein	*L. rhamnosus* GG	98% - 5e-34	YP_003171844.1	_	_
211	AT/AT	240	5S ribosomal RNA	*L. rhamnosus* GG	98% - 2e-11	NR_103302.1	_	_
212	AT/AT	234	5S ribosomal RNA	*L. rhamnosus* GG	98% - 4e-09	NR_103302.1	_	_

^a^When available, EC numbers assigned to the putative enzymatic reactions are provided.

^b^The column indicates the microorganism of the best hit from BLASTX search.

^c^Max identity and E-value from the best hit of BLASTX search are provided.

^d^Pathway assignment was performed according to COG functional categories and KEGG pathway database.

^e^E, Amino acid transport and metabolism; F, Nucleotide transport and metabolism; G, Carbohydrate transport and metabolism; M, Cell wall/membrane/envelope biogenesis; R, General function prediction only.

It is known that plasmids often carry genes that might be essential for survival under harsh conditions, encoding important traits, such as enzymes involved in secondary metabolic pathways [33]. Plasmids are known to be a source of LAB genetic and phenotypic diversity which occasionally confers adaptive advantages to host strains [34]. However, further studies are clearly needed to better explore the role of plasmid sequences in the *L. rhamnosus* adaptation to the cheese ripening environment. To validate the cDNA-AFLP expression profiles, 3 genes, encoding pyruvate oxidase (*spxB*), L-xylulose 5-phosphate 3-epimerase (*ulaE*), and xylulose-5-phosphate phosphoketolase (*xfp*) were selected for qPCR. The relative mRNA abundances were normalized by that of the commonly used reference gene *16S rDNA*, and expressed as a ratio of CB to MRS levels. Amplification efficiency for all assays ranged between 85 and 105%. Confirming the reliability of cDNA-AFLP results, all transcripts were more abundant in CB, with expression ratios over 5-fold (Table 2).

To investigate a possible role for these genes in allowing *L. rhamnosus* growth in cheese during ripening, *in silico* analyses were carried out.

### SpxB

*In silico* analysis of TDF no. 93 (305 bp), encoding 101 amino acid residues, revealed the highest identity in amino acid sequence (93%) with a pyruvate oxidase (SpxB) from *L. rhamnosus* GG (Table 3). Lower levels of identity were observed for SpxB of other members of *L. casei* group (*L. casei*, 79%; *L. paracasei* subsp. *paracasei*, 79%; *L. zeae*, 75%). BLASTX search also returned a number of pyruvate oxidases of other NSLAB, such as *L. curvatus* (55%), *L. buchneri* (46%), *L. brevis* (46%), *L. plantarum* (41%) and *L. pentosus* (41%), as well as of non-*Lactobacillus* bacteria.

**Figure 2:**
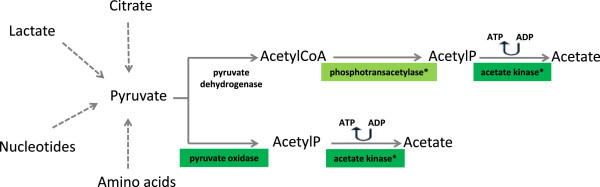
**Conversion of pyruvate into acetate.** Enzymes showing differences in protein (*) or transcript abundance for *L. rhamnosus* PR1019 grown in CB compared to MRS are highlighted. Dark green, expression ratio CB versus MRS 5 to 10; light green, expression ratio CB versus MRS < 5. Transcript data are from the present study. Protein data are from Bove et al. [16].

SpxB is an enzyme involved in the pyruvate metabolism pathway. LAB can metabolize pyruvate into lactate by lactate dehydrogenase (LDH) or into acetate via pyruvate formate lyase (PFL), phosphotransacetylase (PTA) and acetate kinase (ACK), or via pyruvate oxidase (POX) pathway [35]. In the latter, pyruvate is oxidized with the production of hydrogen peroxide and acetyl phosphate, followed by acetate production and ATP generation via ACK (Figure 2).

To our knowledge, this is the first evidence of activation of the POX pathway in *L. rhamnosus*. On the contrary, POX activity has been extensively described to date in *L. plantarum* and involved with acetate production in its survival during the stationary phase of aerobic growth [35-39]. In particular, accumulation of acetate instead of lactate is thought to play a role in ensuring the pH homeostasis with an overall beneficial effect for the cell [37,40]. The additional ATP generated via ACK has been shown to enhance the biomass production [41]. Interestingly, Lorquet et al. [37] showed that in the late stationary phase, when the production of acetate stopped, an OD decrease resulting from lytic processes occurred. The hypothesis is that in the absence of ATP production, protons can no longer be extruded by ATPases with a consequent dissipation of the proton motive force, which has been shown to be one of the mechanisms triggering autolysis of gram-positive bacteria.

Interestingly, high levels of acetic acid and low levels of lactic acid have been recently observed in *L. rhamnosus* strains grown in CB under the same conditions of our study [16,42] Furthermore, by a proteomic approach, Bove et al. [16] showed an increase in expression of PTA and ACK, which are involved in the synthesis of acetic acid in a branch of the pyruvate metabolism other than POX pathway (Figure 2), during *L. rhamnosus* growth in CB compared to MRS. Highlighting a possible alternative route of degradation of pyruvate to acetate (the POX pathway; Figure 2), our transcriptomic results seem to complement data from proteomics, strengthening the hypothesis that *L. rhamnosus* can utilize pyruvate as a growth substrate during cheese ripening.

Pyruvate is an intracellular metabolite that could be produced through different metabolic routes using the carbon sources present in cheese (i.e. through metabolism of citrate, lactate, amino acids, and nucleotides). Moreover, pyruvate can be released in the cheese matrix with starter lysis. Liu et al. [43] showed that the activity of POX in *L. plantarum* could be related to the catabolism of L-serine. According to the authors, L-serine is deaminated via a serine dehydratase into pyruvate, which is subsequently converted into acetate by the POX enzyme [43]. Pyruvate conversion by POX has been recently supposed also in *L. casei*[44]. Looking at the closely related taxonomic groups of these heterofermentative lactobacilli and considering that they share similar ecological niches, including ripened cheese, it appears interesting to further investigate in the future the role of pyruvate metabolism during cheese ripening.

**Figure 3:**
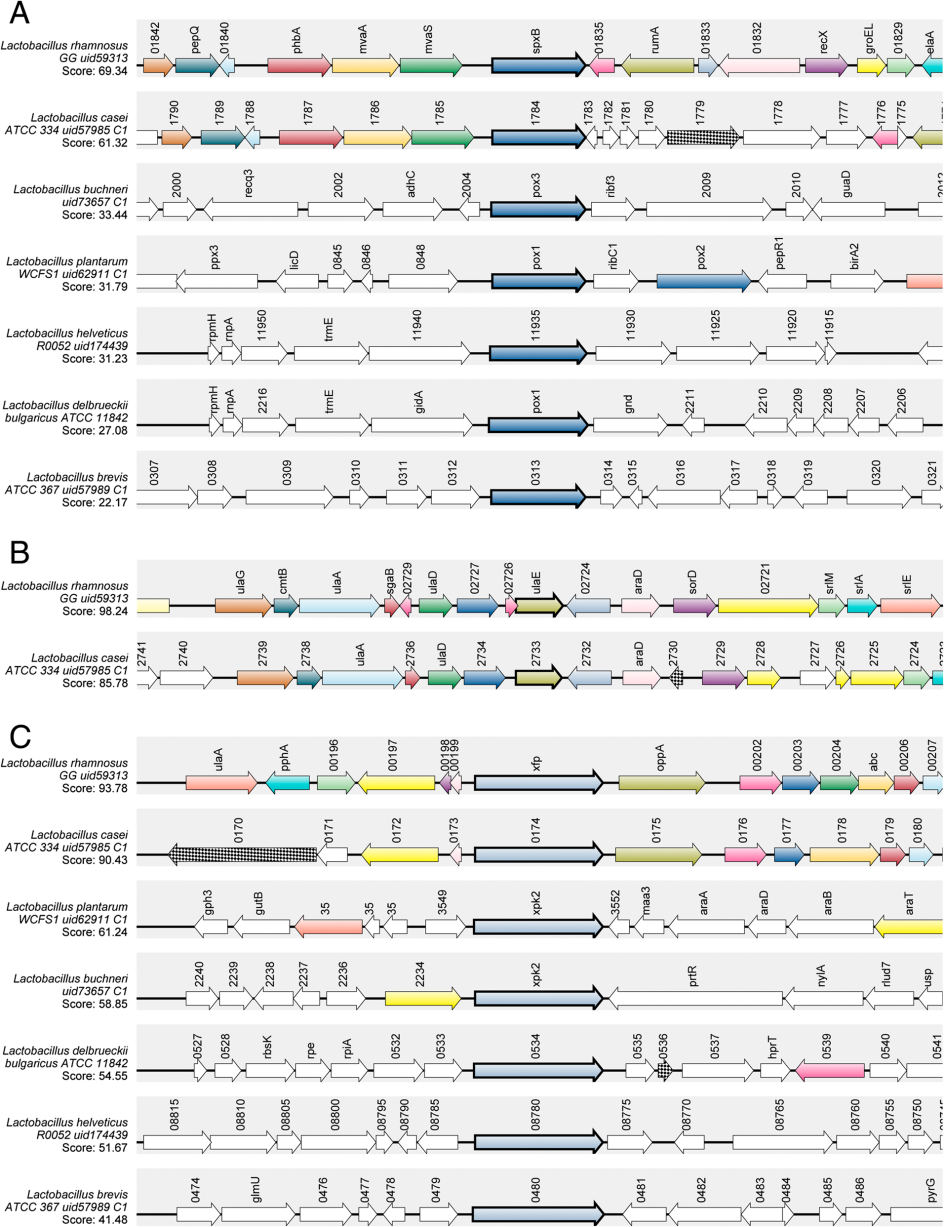
**Schematic diagram for genome regions surrounding spxB, ulaE and xfp locus in diverse lactobacilli. (A)**, *spxB*. **(B)**, *ulaE*. **(C)**, *xfp*. Gene syntenies were explored using the web service SyntTax [27]. TDF-derived protein sequences were used to query the selected genomes. Genes corresponding to query proteins are drawn in bold. A consistent color coding allows identification of orthologs and paralogs. Some gene names are indicated. Normalized BLAST scores are visualized. Reference organisms: *L. rhamnosus* GG, *L. casei* ATCC 334, *L. buchneri* CD034, *L. plantarum* WCFS1, *L. helveticus* R0052, *L. delbrueckii* subsp. *bulgaricus* ATCC 11842 *and L. brevis* ATCC 367. For *ulaE*, a conserved gene order was observed only in *L. rhamnosus* GG and *L. casei* ATCC 334.

**Figure 4:**
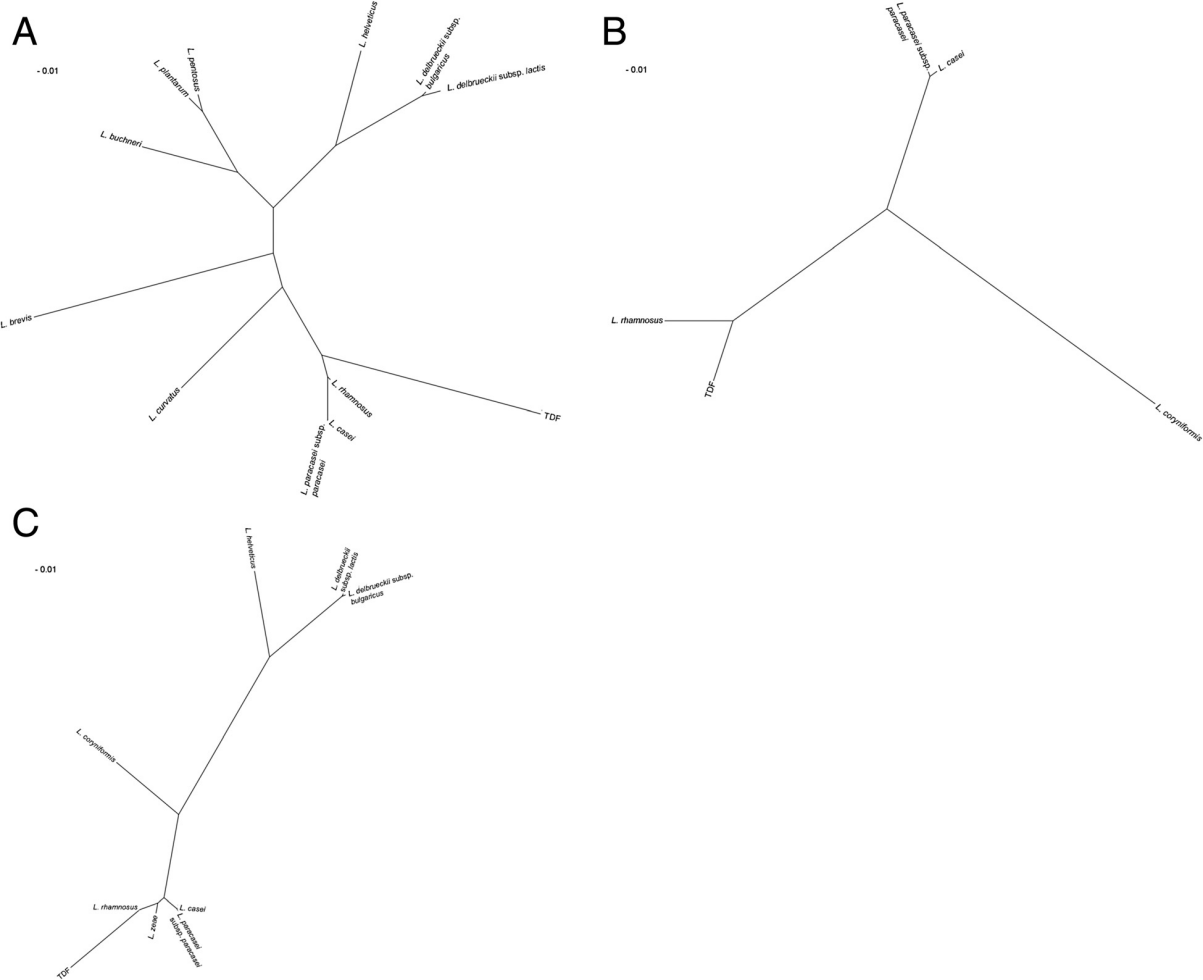
**Unrooted phylogram tree of spxB, ulaE and xfp sequences from diverse lactobacilli. (A)**, spxB. **(B)**, ulaE. **(C)**, xfp. Protein alignments were performed using ClustalW2 [30] and used for phylogenetic tree construction at the Interactive Tree of Life [31]. Reference organisms: *L. rhamnosus* GG, *L. casei* ATCC 334, *L. paracasei* subsp. *paracasei* ATCC 25302, *L. zeae* (accession no. WP_010489923.1), *L. buchneri* CD034, *L. plantarum* WCFS1, *L. helveticus* R0052, *L. delbrueckii* subsp. *lactis* DSM 20072, *L. delbrueckii* subsp. *bulgaricus* ATCC 11842, *L. curvatus* CRL 705, *L. brevis* ATCC 367, *L. pentosus* KCA1, *L. coryniformis* (ulaE, accession no. WP_010012151.1; xfp, WP_010012483.1).

Notably, 3 genes encoding putative pyruvate oxidases are harbored in the completely sequenced genomes of *L. rhamnosus* GG and *L. casei* ATCC 334, whereas 4 and 5 *pox* genes were retrieved in the genome sequences of *L. buchneri* CD034 and *L. plantarum* WCFS1, respectively. Goffin et al. [36] reported that among the predicted *pox* genes encoded in the *L. plantarum* lp80 genome, only *poxB* and *poxF* appeared to be involved in the generation of acetate from lactate during the stationary phase of aerobic growth. Interestingly, *poxB* and *poxF* genes shared 63 and 61% amino acid similarity with TDF 93, respectively. To date, only one gene potentially encoding for pyruvate oxidase has been located in the complete genome sequences of the SLAB *L. helveticus* R0052 and *L. delbrueckii* subsp. *bulgaricus* ATCC 11842. The pyruvate oxidase gene of *L. rhamnosus* GG with the highest homology to TDF 93 is flanked by genes whose order and transcriptional orientation are partially shared with *L. casei* ATCC 334 but not with *L. buchneri* CD034, *L. plantarum* WCFS1, *L. helveticus* R0052, *L. delbrueckii* subsp. *bulgaricus* ATCC 11842 and *L. brevis* ATCC 367 (Figure 3A). In particular, *spxB* locus in *L. rhamnosus* and *L. casei* genomes is preceded by three genes encoding putative hydroxymethylglutaryl-CoA synthase, hydroxymethylglutaryl-CoA reductase and acetyl-CoA acetyltransferase. These enzymes are known to be involved in the mevalonate pathway, routing acetyl-CoA towards isoprenoid biosynthesis. However, whether these proteins are actually expressed in *L. rhamnosus* and play a role in deviating the flow of acetyl-CoA from the acetate production via PTA and ACK during cheese ripening still remain to be determined. According to PePPER, *spxB* gene from *L. rhamnosus* GG was predicted to be monocistronically transcribed. Phylogenetic tree showed a clear segregation of putative pyruvate oxidases from *L. casei* group (Figure 4A). As expected, a subgroup was represented by POX proteins from the SLAB *L. helveticus*, *L. delbrueckii* subsp. *bulgaricus* and *L. delbrueckii* subsp. *lactis. L. plantarum* and *L. pentosus* homologues clustered together and close to *L. buchneri*. Multiple sequence alignment of TDF 93 and pyruvate oxidase protein sequences from several NSLAB and SLAB is shown in Additional file 1: Figure S1A.

### UlaE

BLASTX analysis of TDF no. 86 (109 bp), putatively encoding 36 amino acid residues, showed the maximum identity (94%) to a protein annotated as L-xylulose 5-phosphate 3-epimerase (ulaE) from *L. rhamnosus* GG (Table 3). Eighty-four percent of identity was exhibited to the same putative protein from other *L. casei* group members (*L. casei* and *L. paracasei* subsp. *paracasei*). Homologues were also found in NSLAB known to play a role in flavor generation and other ripening processes: *L. suebicus* (74%), *L. coryniformis* (72%) and *Carnobacterium maltaromaticum* (69%).

UlaE is an epimerase involved with other enzymes (UlaD and UlaF) in the production of D-xylulose 5-phosphate [45,46], an intermediate in the pentose phosphate pathway.

According to SyntTax, regions up and downstream of *ulaE* gene from *L. rhamnosus* GG shared a conserved gene order with *L. casei* ATCC 334, whereas no synteny was found in *L. buchneri* CD034, *L. plantarum* WCFS1, *L. helveticus* R0052, *L. delbrueckii* subsp. *bulgaricus* ATCC 11842 and *L. brevis* ATCC 367 genomes (Figure 3B). According to PePPER analysis of *L. rhamnosus* GG genome, a potential terminator stem-loop structure was identified 82 bp downstream from the *araD* gene stop codon. No putative promoters were predicted up to 5000 bp upstream of *ulaE* gene. Interestingly, the upstream LGG_02727 gene was annotated as a transcriptional regulator, belonging to DeoR family. Phylogenetic analysis of L-xylulose 5-phosphate 3-epimerase homologues revealed that ulaE predicted protein from *L. rhamnosus* clustered close to the putative enzymes from other *L. casei* group members and *L. coryniformis* (Figure 4B). Multiple sequence alignment of TDF 86 and homologs from several NSLAB is shown in Additional file 1: Figure S1B.

### Xfp

TDF no. 40 (302 bp) displayed the highest identity (99%) in amino acid sequence with a putative phosphoketolase (xfp) from *L. rhamnosus* GG (Table 3). Percentages of identity > 95% were found with other *L. casei* group members (*L. zeae*, 98%; *L. paracasei* subsp. *paracasei*, 96%; *L. casei*, 96%). BLASTX search also revealed a significant match to a predicted xylulose-5-phosphate phosphoketolase from *L. coryniformis* (identity 75%). Interestingly, lower levels of identity were obtained with SLAB, such as *L. delbrueckii* subsp. *bulgaricus* (56%), *L. delbrueckii* subsp. *lactis* (56%) and *L. helveticus* (55%).

**Figure 5:**
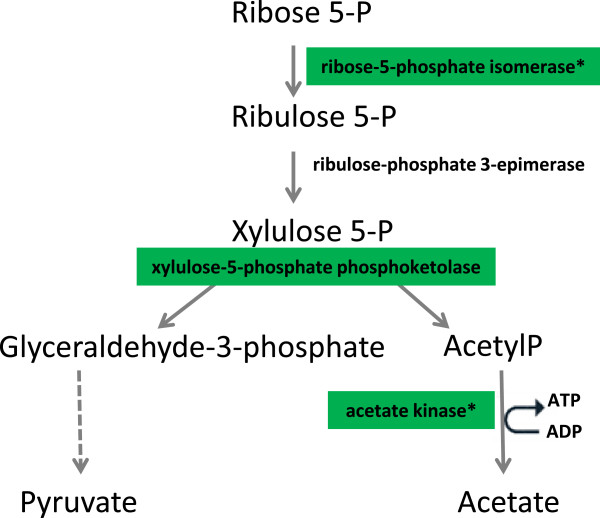
**Degradation of ribose.** Enzymes showing differences in protein (*) or transcript abundance for *L. rhamnosus* PR1019 grown in CB compared to MRS are highlighted. Dark green, expression ratio CB versus MRS 5 to 10. Transcript data are from the present study. Protein data are from Bove et al. [16].

Facultatively heterofermentative LAB, like *L. rhamnosus*, degrade hexoses via the Embden-Meyerhoff-Parnas pathway and pentoses via the phosphoketolase pathway (PKP). Xylulose 5-phosphate phosphoketolase is the central enzyme of PKP. In the presence of inorganic phosphate this enzyme converts xylulose 5-phosphate into glyceraldehyde 3-phosphate and acetylphosphate (Figure 5) [47]. Recently, McLeod et al. [48] studied the transcriptome response of *L. sakei* during growth on ribose, demonstrating that the ribose uptake and catabolic machinery are highly regulated and closely linked with the catabolism of nucleotides. It is known that ribonucleosides are source of ribose as a fermentable carbohydrate in environments where free carbohydrates are lacking. For example, in the meat, a rich environment but carbohydrate-poor substrate for microorganisms, the ability of *L. sakei* to use nucleosides offers a competitive advantage [49]. Nucleosides represent a potential energy source also in the cheese environment, where microbial autolysis occurs, releasing ribose- and desoxyribose-containing nucleic acids [14]. Notably, it has been observed that ribose released after lysis of SLAB decreased steadily in parallel with the growth of facultatively heterofermentative lactobacilli, strongly suggesting that these bacteria used ribose as a growth substrate [14].

The over-expression of *xfp* mRNA levels in *L. rhamnosus* grown in CB, as found in our study, seems to support this hypothesis. Moreover, our findings are in agreement with the proteomic data of Bove and colleagues [16], who observed an increase in expression level of ribose-5-phosphate isomerase (Rpi) after *L. rhamnosus* growth in CB compared to MRS. This enzyme acts in a step upstream of xfp in the pathway that leads from ribose 5-phosphate (R5P) to the production of acetate, catalyzing the conversion of R5P to ribulose 5-phosphate (Figure 5).

According to Pfam search, TDF 40-deduced 100 amino acid sequence contains a portion of the XFP C-terminal domain (pfam09363). The genetic organization and location of *xfp* gene on *L. rhamnosus* GG and *L. casei* ATCC 334 chromosomes were shown to be highly similar (Figure 3C). In particular, *xfp* genes are preceded by a divergently transcribed ORF, encoding a major facilitator superfamily transporter, and are followed by several genes predicted to encode components of ABC transporter and PTS systems for sugar uptake. According to PePPER, no high-scoring promoter consensus sequences were identified in the 5000-bp upstream region of *xfp* gene in *L. rhamnosus* GG. A Rho-independent transcriptional terminator was predicted to occur 43 bp downstream from the *xfp* stop codon. Phylogram showed that xfp proteins from *L. casei* group made a separate cluster, close to the putative enzyme from *L. coryniformis* (Figure 4C). Analogously, different clusters were observed for the SLAB *L. helveticus*, *L. delbrueckii* subsp. *lactis* and *L. delbrueckii* subsp. *bulgaricus*. Additional file 1: Figure S1C displays a multiple sequence alignment of TDF 40 and putative phosphoketolases from several SLAB and NSLAB.

## Conclusions

In this study, we applied a transcriptomic approach, based on cDNA-AFLP and qPCR, to investigate the physiological adaptation of *L. rhamnosus* to the cheese environment. *L. rhamnosus* is known to be one of the few NSLAB species able to survive and grow during long ripening of sseveral cheeses. In particular, the strain *L. rhamnosus* PR1019, isolated from 4-month-ripened PR cheese, has previously shown a great ability to growth in CB coupled with high levels of production of acetic acid. By comparing the gene expression profiles of *L. rhamnosus* PR1019 in CB respect to MRS, we identified among others as over-expressed in CB, genes linked to the conversion of pyruvate to acetate as well as to the pathway of ribose degradation. Notably, the activation of POX pathway in *L. rhamnosus* has never been observed before.

Pyruvate is a intracellular metabolite that could be produced by different metabolism using the carbon source present in cheese and can be released in the cheese matrix with the starter lysis. Similarly the ribonucleosides release with starter lysis could be carriers of ribose that represents a fermentable carbohydrate in an environments such cheese where carbohydrates are lacking.

Both pyruvate degradation and ribose catabolism induce a metabolite flux toward acetate, coupled with ATP production via acetate kinase. Taking into account these consideration, and in agreement with previous findings [16] we assume that *L. rhamnosus* when growing in media poor in carbohydrates, such as CB, arguably uses different metabolic pathways to produce energy. Notably, the transcriptomic approach employed in this study evidenced the over-expression in CB of enzymes other than those identified through proteomics by Bove et al. [16], acting at different steps or in different branches of the ribose and pyruvate utilization pathways. This discrepancy, probably owing to issues of technique sensitivity and resolution, highlighted the need to integrate transcriptomic and proteomic data in order to get a view as complete as possible of the *L. rhamnosus* metabolic adaptations during cheese ripening.

Since, to our knowledge, this is the first study that showed the activation of POX pathway in *L. rhamnosus*, further work will be directed to investigate more in depth the role of the pyruvate metabolism in the growth of this specie in cheese.

## Abbreviations

LAB: Lactic acid bacteria; SLAB: Starter lactic acid bacteria; NSLAB: Non-starter lactic acid bacteria; L.: *Lactobacillus*; cDNA-AFLP: cDNA-amplified fragment length polymorphism; qPCR: Quantitative real-time reverse transcription-PCR; CB: Cheese broth; TDFs: Transcript-derived fragments; SpxB/POX: Pyruvate oxidase; UlaE: L-xylulose 5-phosphate 3-epimerase; Xfp: xylulose-5-phosphate phosphoketolase.

## Competing interests

The authors declare that they have no competing interests.

## Author’s contributions

CL: conceived the study, participated in its coordination and drafted the manuscript. ST: carried out genetic and bioinformatic analysis and helped to draft the manuscript. AM: carried out genetic analysis, participated in data collection and their interpretation. ES: carried out genetic analysis, participated in data collection and their interpretation. EN: critically revised the paper. PB: critically revised the paper. MG: conceived the study, supervised the research work and critically revised the paper. All authors read and approved the final manuscript.

## Supplementary Material

Additional file 1: Figure S1 Multiple sequence alignment of spxB (A), ulaE (B) and xfp (C) sequences from diverse lactobacilli. Conservation plots and consensus sequences are shown at the bottom. Protein alignments were performed and represented using CLC-Bio sequence viewer [32]. Reference organisms: *L. rhamnosus* GG, *L. casei* ATCC 334, *L. paracasei* subsp. *paracasei* ATCC 25302, *L. zeae* (accession no. WP_010489923.1), *L. buchneri* CD034, *L. plantarum* WCFS1, *L. helveticus* R0052, *L. delbrueckii* subsp. *lactis* DSM 20072, *L. delbrueckii* subsp. *bulgaricus* ATCC 11842, *L. curvatus* CRL 705, *L. brevis* ATCC 367, *L. pentosus* KCA1, *L. coryniformis* (ulaE, accession no. WP_010012151.1; xfp, WP_010012483.1). Click here for file
