# Supplementary figures and images for: Transcriptomic clues to understand the growth of Lactobacillus rhamnosus in cheese
Source: BMC Microbiol. 2014 Feb 7;14:28. doi: 10.1186/1471-2180-14-28 (PMC3928093; doi:10.1186/1471-2180-14-28)

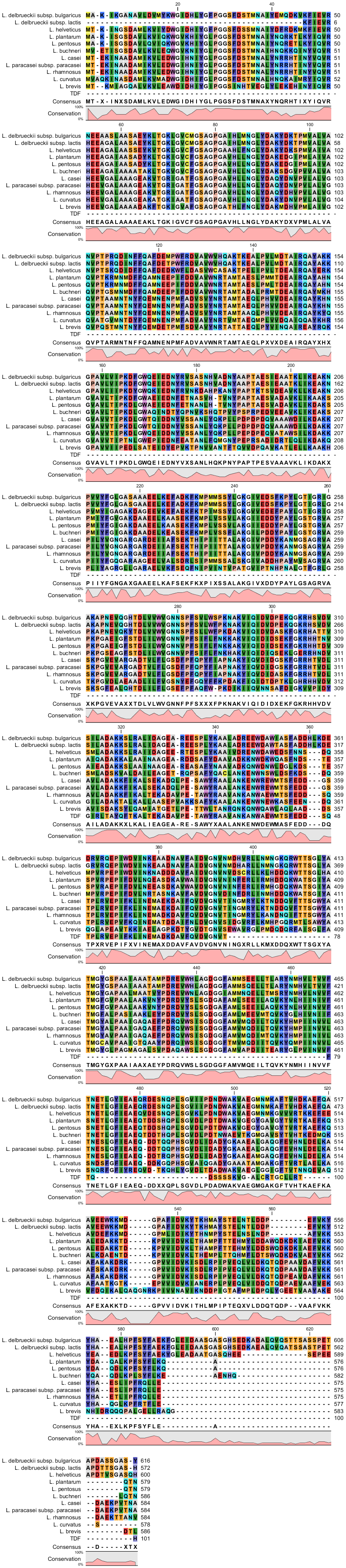

Supplement: Additional file 1: Figure S1 — Multiple sequence alignment of spxB (A), ulaE (B) and xfp (C) sequences from diverse lactobacilli. Conservation plots and consensus sequences are shown at the bottom. Protein alignments were performed and represented using CLC-Bio sequence viewer [32]. Reference organisms: L. rhamnosus GG, L. casei ATCC 334, L. paracasei subsp. paracasei ATCC 25302, L. zeae (accession no. WP_010489923.1), L. buchneri CD034, L. plantarum WCFS1, L. helveticus R0052, L. delbrueckii subsp. lactis DSM 20072, L. delbrueckii subsp. bulgaricus ATCC 11842, L. curvatus CRL 705, L. brevis ATCC 367, L. pentosus KCA1, L. coryniformis (ulaE, accession no. WP_010012151.1; xfp, WP_010012483.1). [file 1471-2180-14-28-S1.zip › 1535713233109022_add1A.tiff]

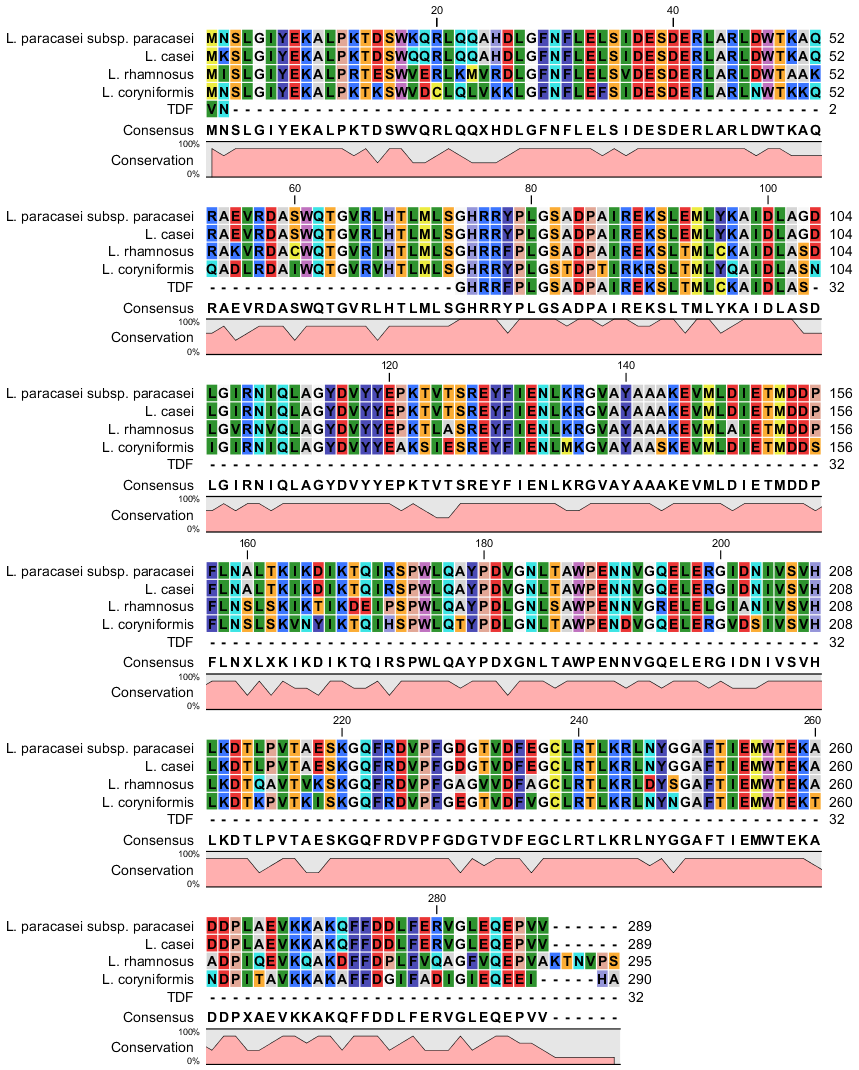

Supplement: Additional file 1: Figure S1 — Multiple sequence alignment of spxB (A), ulaE (B) and xfp (C) sequences from diverse lactobacilli. Conservation plots and consensus sequences are shown at the bottom. Protein alignments were performed and represented using CLC-Bio sequence viewer [32]. Reference organisms: L. rhamnosus GG, L. casei ATCC 334, L. paracasei subsp. paracasei ATCC 25302, L. zeae (accession no. WP_010489923.1), L. buchneri CD034, L. plantarum WCFS1, L. helveticus R0052, L. delbrueckii subsp. lactis DSM 20072, L. delbrueckii subsp. bulgaricus ATCC 11842, L. curvatus CRL 705, L. brevis ATCC 367, L. pentosus KCA1, L. coryniformis (ulaE, accession no. WP_010012151.1; xfp, WP_010012483.1). [file 1471-2180-14-28-S1.zip › 1535713233109022_add1B.tiff]

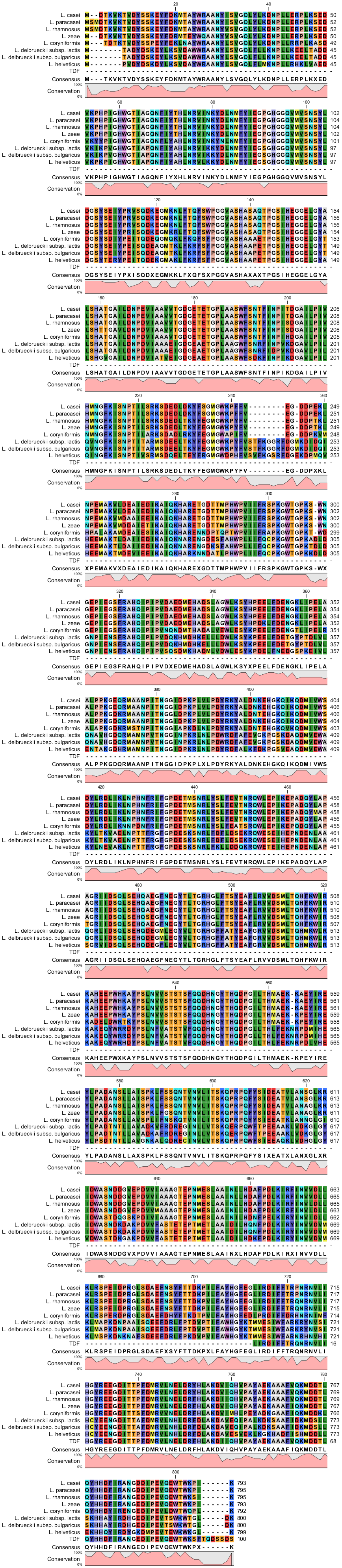

Supplement: Additional file 1: Figure S1 — Multiple sequence alignment of spxB (A), ulaE (B) and xfp (C) sequences from diverse lactobacilli. Conservation plots and consensus sequences are shown at the bottom. Protein alignments were performed and represented using CLC-Bio sequence viewer [32]. Reference organisms: L. rhamnosus GG, L. casei ATCC 334, L. paracasei subsp. paracasei ATCC 25302, L. zeae (accession no. WP_010489923.1), L. buchneri CD034, L. plantarum WCFS1, L. helveticus R0052, L. delbrueckii subsp. lactis DSM 20072, L. delbrueckii subsp. bulgaricus ATCC 11842, L. curvatus CRL 705, L. brevis ATCC 367, L. pentosus KCA1, L. coryniformis (ulaE, accession no. WP_010012151.1; xfp, WP_010012483.1). [file 1471-2180-14-28-S1.zip › 1535713233109022_add1C.tiff]
